# Neurovascular bundle sparing in hypofractionated radiotherapy maintained with realistic treatment uncertainties

**DOI:** 10.1016/j.phro.2025.100714

**Published:** 2025-01-30

**Authors:** Roel C. Kwakernaak, Victor J. Brand, Jesús Rojo-Santiago, Femke E. Froklage, Mischa S. Hoogeman, Steven J.M. Habraken, Maaike T.W. Milder

**Affiliations:** Erasmus MC Cancer Institute University Medical Center Rotterdam Department of Radiotherapy the Netherlands

## Abstract

**Background and purpose:**

Erectile dysfunction is a common side effect of radiotherapy for prostate cancer. To mitigate this toxicity, it has been suggested to limit the dose to critical nerves and vessels. We investigated the feasibility of sparing the neuro-vascular bundles (NVBs) in stereotactic body radiotherapy under the impact of realistic treatment uncertainties.

**Materials and methods:**

Non-sparing and sparing NVB treatment plans, delivered in 5 × 7.25 Gy, were automatically generated for 20 patients. Polynomial Chaos Expansion (PCE) was used to fast and accurately model the dose against treatment errors. PCE enabled a robustness evaluation of 100.000 treatment scenarios per plan, allowing to derive scenario distributions of clinically relevant dose volume histogram parameters and population dose histograms.

**Results:**

An average decrease of 3.7 Gy and 4.4 Gy in the median $D_{0.1cm^{3}}$ of the NVB was achieved in the patient population in the presence of realistic treatment uncertainties for non-coplanar (NC) and coplanar (C) plans respectively. Sparing NVBs decreased planning target volume coverage by 2.1 % in $V_{36.25Gy}$ on average, however clinical target volume (CTV) dose remained adequate. Population dose histograms showed that, while sparing does impact dose volume histogram parameters of organs at risk (OARs), the probability of a scenario exceeding planning constraints was limited.

**Conclusion:**

NVB sparing was maintained in the presence of treatment uncertainties without compromising CTV coverage or OAR dose. There was no significant difference in the achieved NVB dose between NC and C plans. The clinical impact of the achieved sparing is subject of ongoing clinical trials.

## Highlights

• Neurovascular bundle sparing maintained with realistic treatment uncertainties.
• Adequate clinical target volume coverage with neurovascular bundle sparing.
• Realistic simulation of treatment uncertainty using polynomial chaos expansion.
• Statistically accurate impact of treatment uncertainties in hypofractionation.

## 1 Introduction

Hypofractionated stereotactic body radiotherapy (SBRT), combines a high fraction dose with a reduced number of fractions enabled by high-precision irradiation. Because of the low α/β of prostate cancer [1], SBRT has shown promising outcomes in the treatment of localized prostate cancer [2], [3], [4]. It utilizes high dose conformity and steep dose gradients to deliver maximum dose to the target while minimizing the dose to organs at risk (OAR) to reduce the chance of adverse effects [5]. Different beam arrangements can be used to achieve these steep dose gradients. Coplanar only utilizes beams from within a single axial plane while non-coplanar uses more degrees of freedom with beams deriving from multiple different angles. In addition, using the combination of SBRT and non-coplanar beam arrangements, better OAR sparing can be achieved compared to coplanar beams for prostate cancer [6].

Erectile dysfunction (ED), is one of the major side-effects of radiotherapy (RT) and significantly impacts quality of life [7]. Commonly, ED is associated with damage incurred to multiple structures linked to erectile function: the penile bulb, crura, internal pudendal artery (IPA) and neuro-vascular bundles (NVBs) [7], [8]. For the NVBs in particular, there is some evidence suggesting that preserving the NVBs may be essential in mitigating both short- and long-term ED [9], [10], [11] as well as urinary incontinence [12], [13]. Optimal results in surgery were found with bilateral NVB sparing [14]. Simultaneously, much is still unknown about the required level of NVB sparing, especially when compared to sparing the penile bulb, crura and IPA, which have been subject of multiple clinical studies [15], [16], [17]. There are ongoing trials using CT-guided and MRI-guided techniques [18], [19] investigating the relationship between dose to the NVB and the onset of toxicity as this relationship remains largely unknown.

Multiple factors complicate the sparing of the NVBs. The NVB anatomy varies widely from patient to patient [20], [21], [22], and MRI scans are required for an accurate delineation [23]. Furthermore, the NVB often overlap with the planning target volume (PTV). The effect of realistic registration and delineation errors and additional treatment delivery uncertainties such as patient- and beam-alignment on the achievable sparing of the NVB in the delivered treatment is still unknown.

In this paper, we report the feasibility of sparing the NVBs in stereotactic body radiotherapy under the impact of realistic treatment uncertainties.

## 2 Materials and methods

### 2.1 Patient data

**Fig. 1:**
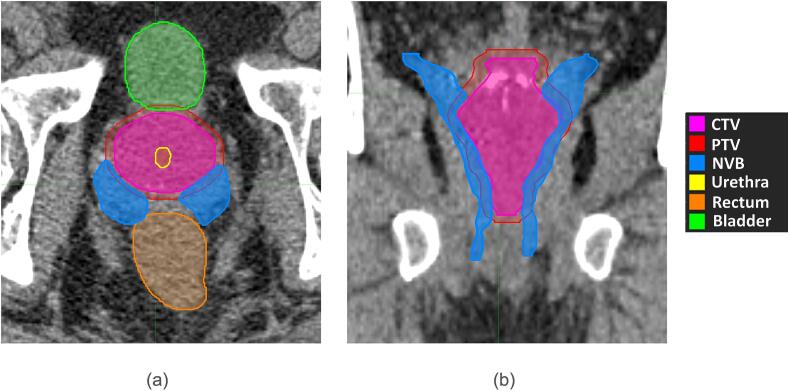
(a) axial and (b) coronal CT slice of a patient with the delineated CTV, PTV, NVB bundles and surrounding OARs.

In this study, 20 patients previously treated at our center for localized prostate cancer, were included. Written informed consent was obtained for all patients. All patients had a T2- weighted MRI sequences (1.5 Tesla) and a radiotherapy planning CT scan (CT voxel size 0.98 × 0.98 × 1.5 mm^3^). The MRI and CT scan were matched aided by the intra-prostatic urethra, clearly visible on both CT- and MRI-scans as a Foley catheter was in situ. The NVB and the prostate were delineated on the MRI-scan under guidance of an experienced radiologist. These contours were subsequently edited, if necessitated by changes in anatomy between MRI and CT, based on anatomic boundaries (e.g. structures overlapping bone) in the CT scan. An example of the anatomy can be found in Fig. 1. The remaining organs-at-risk were delineated on the CT-scan.

### 2.2 Treatment planning

Retrospective treatment plans were generated according to the PACE-trial protocol with patients receiving 5 fractions of 7.25 Gy using a 3 mm CTV-PTV margin [24]. No clinically validated constraint for the NVB is known yet. However, as the NVBs can be considered to be serial organs, the focus of sparing was the near maximum dose ($D_{0.1{cm}^{3}}$), as done for similar structures such as the brachial or sacral plexus [25]. Pareto optimal treatment plans were generated using Erasmus iCycle [26], an in-house created algorithm for prioritized multicriteria optimization of radiotherapy treatment plans. In total, four plans were generated, two non-sparing and two sparing plans, with a coplanar (C) and non-coplanar (NC) beam arrangements, respectively. The latter previously shown to be advantageous in prostate treatments were employed [6]. Plans for C and NC beam geometries were generated using two respective wishlists consisting of optimization constraints and objectives, which were optimized to generate treatment plans according to the PACE-constraints. Plans were generated with a maximum dose of 133 % or 48 Gy, which is in correspondence with a prescription isodose line of 75 %. To generate the sparing plans, one objective was added to minimize the dose to both NVB, primarily focused to minimize the near-max dose. This NVB objective was then not considered to generate the non-sparing plans. For NC plans, a beam angle class-solution was used, based on a previous publication [27]. For the C plans, 72 equi-angular beam orientations in the axial plane through the isocenter were used, covering 360° around the patients with an angular separation of 5° [6].

### 2.3 Polynomial chaos expansion

**Fig. 2:**
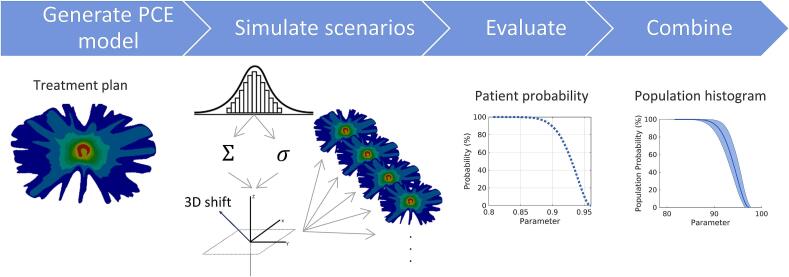
Workflow of the PCE simulation: dose distributions are calculated based on a 3D geometrical shift obtained from sampling from the random and systematic error which are both sampled from a Gaussian distribution. By simulating these fractionated treatments DVH parameters can be statistically assessed using the different PCE percentiles, the entire patient population can be analyzed using population histograms.

Polynomial Chaos Expansion (PCE) was used to model the dependence of treatment uncertainties on the 3D dose distribution, which has already been used in both conventional radiotherapy and proton therapy [28], [29], [30]. PCE provides a fast and computationally efficient patient- and treatment plan-specific model of the dose engine, enabling the simulation and calculation of the dose for 100.000 complete fractionated treatments. Each fractionated treatment was simulated by assuming a systematic (Σ) error for each complete treatment and incorporating a random (σ) error for each fraction. For each fractionated treatment, systematic and random errors had a geometrical component in the x-,y- and z-plane, resulting in the simulation of different realistic error scenarios. This enables a fast and an accurate simulation of a high number of scenarios with proper statistical weighting and the derivation of probability distributions of clinically relevant DVH parameters. Fig. 2 schematically represents the workflow. For each plan, a PCE model was constructed.

PCE has been implemented and validated for radiotherapy in MATLAB (version 2021b) [31]. PCE uses multi-dimensional Hermite polynomials in combination with expansion coefficient to determine the dose $D_{i}$ in each voxel i with error $\vec{\xi }$, which is a stochastic value retrieved from a probability density function:

(2) $$D_{i}\left(\vec{\xi }\right)=\sum_{k=0}^{P}a_{i,k}\Psi_{k}\left(\vec{\xi }\right)$$

where $\Psi_{k}$ is a multi-dimensional Hermite polynomial and the $a_{i,k}$ expansion coefficients, which are determined using linear regression. The polynomial order was chosen to be 5, for an optimal balance between computational efficiency and accuracy. The underlying theory supporting PCE is outside of the scope of this research but can be reviewed in previous publications [30], [32].

### 2.4 Treatment uncertainties

To obtain (1SD) systematic and random treatment errors consistent with a PTV margin of 3 mm [33], [34], [35], [36], Van Herk’s margin recipe [37] was employed. The distribution of the systematic and random errors where considered equal, referring to [38] and the references therein. Therefore, the systematic and random error sizes in the x, y and z directions were determined:

(1) $$m_{\mathrm{ptv}}=2.5\Sigma +0.7\sigma =3mm$$

so that values for Σ=σ=0.94 mm.

### 2.5 Scenario dose calculation with PCE

The systematic and random errors for each scenario were sampled from a Gaussian distribution, *w*ith a mean of zero and standard deviation of sigma*,* independently for the x,y, and z dimensions. This resulted in a realistic distribution of treatment uncertainties. The dose distribution for each scenario was calculated using PCE, in addition to the nominal scenario, i.e. the scenario without the presence of any errors. Thus, we simulated and calculated the dose for 100.000 complete fractionated treatments.

### 2.6 Data analysis

For each simulated treatment, clinically relevant DVH parameters were calculated for the OARs, PTV, and the clinical target volume (CTV). PCE distribution were obtained for these parameters from the 100.000 different simulated dose distributions for individual patients. These PCE distribution were used to create individual probability distributions for each patient. The simulated dose distributions were used to create the probabilistic DVHs.

To evaluate the impact of NVB sparing on the dose distributions for our patient cohort, population histograms were created for the NVB, PTV, CTV, urethra, rectum and bladder. These histograms, computed as the population mean of individual patient probability distributions [28], probabilistically evaluate the dose distribution for all simulated treatments. Using these population histograms, DVH parameters were compared to the constraints used in the PACE-trial. The median (50th percentile) and the 5th and 95th percentiles from the population histograms were reported. Statistics to compare plans were calculated using a T-student test. For a p-value higher than 0.3 distributions were considered significantly equal, for values lower than 0.05 distributions were considered significantly different.

## 3 Results

It was observed that the mean of the of the $D_{0.1{cm}^{3}}$ distribution of the NVB and the nominal value were approximately equal, indicating that the expected value of the distribution was roughly equal to the nominal value observed in planning (see Fig. A.1 in the supplementary materials).

**Fig. 3:**
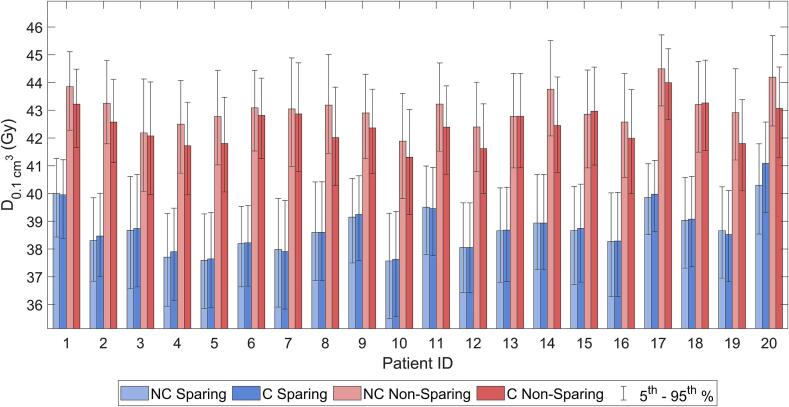
D_{0.1 cm^3} of the left NVB for the individual patients. Shown are all 4 treatment plans: C Non-sparing, NC Non-sparing, C Sparing and NC Sparing. The 5th and 95th percentiles from the PCE distributions are plotted.

Fig. 3 shows the sparing in the NVB bundles for all individual patients produced using the PCE models. There was no significant difference (p>0.3) between the $D_{0.1cm^{3}}$ of the left and right NVB. For the non-sparing plans, the nominal value of the $D_{0.1cm^{3}}$ for the population average median was 42.5 Gy and 43.1 Gy for the C and NC plans respectively. Including treatment errors, these values remain unchanged: the population average medians were 42.5 (40.7–44.0, 5th and 95th percentiles of the probability distribution) Gy and 43.1 (41.4–44.4) Gy for C and NC plans respectively. In the sparing plans, using the nominal scenario, the population average median $D_{0.1cm^{3}}$ of the left NVB was reduced by respectively 4.0 Gy and 4.6 Gy to 38.5 Gy for C and NC plans. When considering treatment errors, the amount of sparing achieved was mostly maintained and the population average median $D_{0.1cm^{3}}$ slightly increased only to 38.8 Gy (36.7–40.8) for C plans and 38.7 (35.6–39.8) Gy for NC plans. The values of $D_{0.1cm^{3}}$, $D_{0.5cm^{3}}$, $D_{1.0cm^{3}}$ and $D_{2.0cm^{3}}$ were similar in sparing C and NC plans (p>0.3), however the amount of sparing achieved was slightly larger in NC plans, i.e. 3.7 Gy vs 4.4 Gy for the population average median $D_{0.1cm^{3}}$. Similar results were obtained for the right NVB, indicating that bilateral sparing can be achieved.

**Fig. 4:**
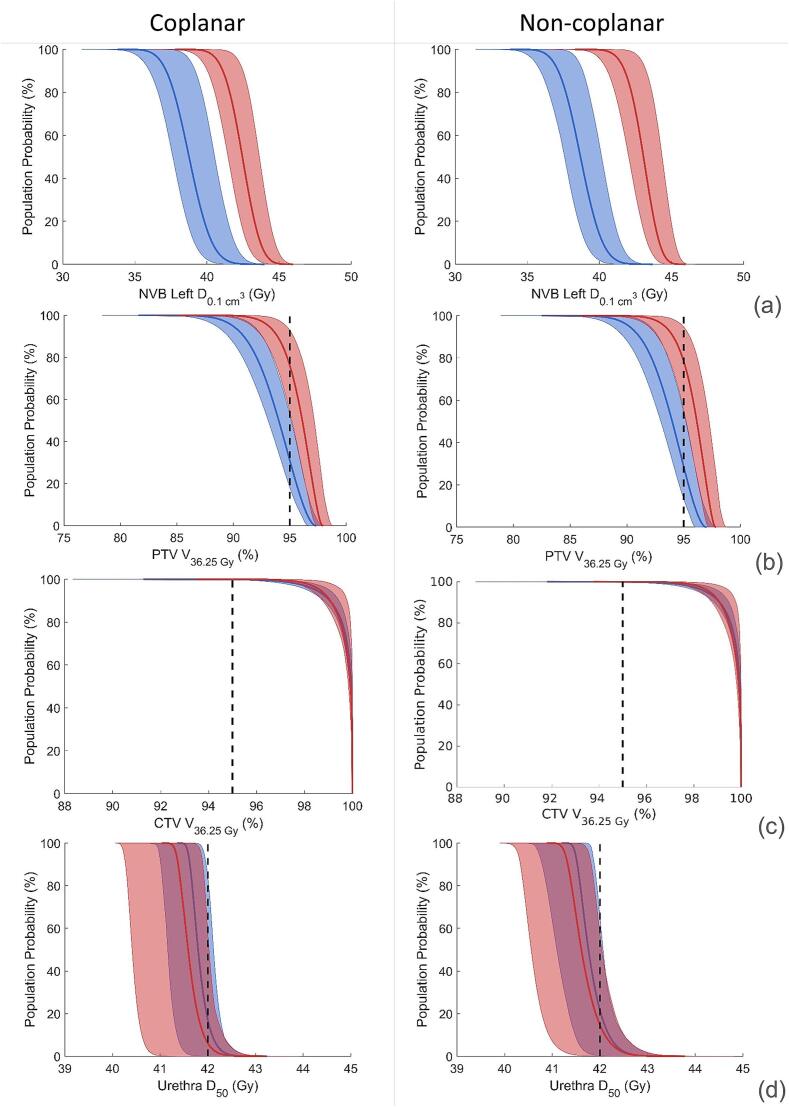
Population dose histogram for coplanar (left) and non-coplanar (right) sparing (blue) and non-sparing (red) treatment plans for (a) NVB Left D_0.1 cm^3^_, (b) PTV-3 mm D_36.25 Gy_, (c) CTV V_36.25 Gy_ and (d) Urethra D_50_. Solid line represent the mean probability, transparent borders show the 5th and 95th percentile of the total values. (For interpretation of the references to colour in this figure legend, the reader is referred to the web version of this article.)

The absence of any overlap in Fig. 4a between the median and the 90 % confidence (from the 5th to the 95th percentiles) show that the difference between sparing and non-sparing plans remains present with realistic treatment errors. Fig. 4b shows that PTV coverage decreased with the impact of treatment errors. For non-sparing plans, the constraint to assess adequate target coverage ($V_{36.25Gy\;,PTV}>95\%$), was not fulfilled for 24.1 % and 21.8 % of the population for C and NC plans respectively. For the sparing plans, this increased to 69.0 % and 71.8 % respectively. The average decrease in $V_{36.25Gy}$ was 2.1 % for both C and NC plans. Despite the loss of PTV coverage, the CTV volume receiving the prescribed dose of 36.25 Gy was not affected by sparing as it remained 100 % in 95 % of the CTV volume for the entire population for all sparing and non-sparing plans, as shown in Fig. 4c. To ensure optimal comparison between non-sparing and sparing plans, the dose distributions for both plans were generated based on one set of optimization parameters, with the NVB constraint disabled in non-sparing optimizations. As a consequence, the PTV coverage was slightly higher in these plans.

The median OAR dose in the population (excluding NVB bundles) increased 1 Gy on average in C and NC plans when NVB sparing was applied, in which no significant difference $\left(p>0.4\right)$ was observed between C and NC beam configurations. However, most values remain well within constraints. The Rectum $D_{2.0cm^{3}}$ constraint was not met when errors were introduced in 0.7 % of the population of non-sparing plans for C and NC (max dose in 90 % confidence interval: 34.3 Gy and 33.9 Gy), this increased to 1.1 % in the sparing plans (max: 34.8 Gy and 34.4 Gy). Urethra dose showed a larger increase when introducing errors: in the non-sparing plans 5.9 % (C) (max: 42.0 Gy) and 14.3 % (NC) (max: 42.3 Gy) of the population did not fulfill the contstraint. Notably, this increased to 16.9 % (max: 42.2 Gy) and 21.6 % (max: 42.3 Gy) patients in the sparing plans. The population dose histogram for the urethra $D_{50}$ can be reviewed in Fig. 4d. An overview of the DVH parameters for the relevant structures in C and NC plans, can be found in Table 1, Table 2.

**Table 1:** Comparison of the nominal scenario and patient probabilities of the Coplanar Sparing and Non-Sparing plans, compared to the planning constraint according to the PACE trial. The 50th, 5th and 95th percentile are reported for the respective DVH parameter.

	PACE constraint	Nominal scenario		Population probability 50 % (95 % −5%)			
		Non-sparing	Sparing	Non-sparing	Met PACE constraint (%)	Sparing	Met PACE constraint (%)
CTV $V_{36.25Gy}$ (%)		99.9	100	99.9 (98.6–100)		99.9 (98.5–100)	
CTV $D_{95}$ (Gy)		41.0	40.2	40.8 (39.5–41.2)		40.0 (38.3–40.8)	
PTV $V_{36.25Gy}$ (%)	>95	97.4	95.1	96.1 (93.1–97.5)	75.9	94.0 (89.9–96.6)	31.0
NVB $LeftD_{0.1cm^{3}}$ (Gy)		42.5	38.5	42.5 (40.7–44.0)		38.8 (36.7–40.8)	
NVB Left $D_{0.5cm^{3}}$ (Gy)		40.9	37.3	40.8 (39.0–42.5)		37.3 (35.1–39.4)	
NVB Left $D_{1.0cm^{3}}$ (Gy)		39.8	36.0	39.7 (37.8–41.3)		36.0 (33.7–38.2)	
NVB Left $D_{2.0cm^{3}}$ (Gy)		37.7	33.3	37.5 (35.6–39.3)		33.2 (30.9–35.6)	
NVB Right $D_{0.1cm^{3}}$ (Gy)		42.4	38.6	42.4 (40.7–44.0)		38.8 (36.8–40.9)	
NVB Right $D_{0.5cm^{3}}$ (Gy)		40.9	37.4	40.9 (39.1–42.5)		37.4 (35.3–39.5)	
NVB Right $D_{1.0cm^{3}}$ (Gy)		39.8	36.2	39.7 (37.9–41.4)		36.2 (34.0–38.3)	
NVB Right $D_{2.0cm^{3}}$ (Gy)		37.8	33.8	37.7 (35.8–39.5)		33.7 (31.4–36.0)	
Urethra $D_{50}$ (Gy)	<42	41.4	41.6	41.6 (41.3–42.0)	94.1	41.8 (41.6–42.2)	83.1
Rectum $D_{50}$ (Gy)	<18.1	4.3	5.9	4.1 (3.7–4.4)	100	5.6 (5.2–6.1)	100
Rectum $D_{20}$ (Gy)	<29	12.4	14.5	12.2 (10.8–13.8)	100	14.3 (12.9–15.9)	100
Rectum $D_{2cm^{3}}$ (Gy)	<36	30.7	31.5	30.6 (26.7–34.3)	99.3	31.3 (27.7–34.8)	98.9
Bladder $D_{10cm^{3}}$ (Gy)	<37	28.6	29.5	28.3 (24.9–31.7)	100	29.2 (25.9–32.5)	100
Bladder $D_{40}$ (Gy)	<18.1	5.5	6.0	5.4 (4.4–6.6)	100	5.9 (4.8–7.2)	100

**Table 2:** Comparison of the Nominal scenario and patient probabilities of the Non-Coplanar Sparing and Non-Sparing plans compared to the planning constraint according to the PACE trial. The 50th,5th and 95th percentile are reported for the respective DVH parameter.

	PACE constraint	Nominal scenario		Population probability 50 % (95 % −5%)			
		Non-sparing	Sparing	Non-sparing	Met PACE constraint (%)	Sparing	Met PACE constraint (%)
CTV $V_{36.25Gy}$ (%)		99.9	100	99.9 (98.7–100)		99.9 (98.5–100)	
CTV $D_{95}$ (Gy)		41.1	40.2	40.9 (39.8–41.3)		40.0 (38.4–40.8)	
PTV $V_{36.25Gy}$ (%)	>95	97.1	95.0	96.1 (93.3–97.5)	78.2	94.0 (90.2–96.3)	28.2
NVB $LeftD_{0.1cm^{3}}$ (Gy)		43.1	38.5	43.1 (41.4–44.4)		38.7 (36.7–40.7)	
NVB Left $D_{0.5cm^{3}}$ (Gy)		41.6	37.2	41.5 (39.6–43.0)		37.2 (35.0–39.2)	
NVB Left $D_{1.0cm^{3}}$ (Gy)		40.3	35.9	40.2 (38.2–41.9)		35.9 (33.4–38.0)	
NVB Left $D_{2.0cm^{3}}$ (Gy)		37.8	32.9	37.6 (35.4–39.6)		32.8 (30.1–35.4)	
NVB Right $D_{0.1cm^{3}}$ (Gy)		43.1	38.8	43.1 (41.3–44.6)		38.8 (36.8–40.8)	
NVB Right $D_{0.5cm^{3}}$ (Gy)		41.6	37.3	41.5 (39.6–43.1)		37.3 (35.2–39.4)	
NVB Right $D_{1.0cm^{3}}$ (Gy)		40.4	36.2	40.2 (38.2–42.0)		36.1 (33.7–38.2)	
NVB Right $D_{2.0cm^{3}}$ (Gy)		38.0	33.4	37.8 (35.6–39.8)		33.3 (30.6–35.7)	
Urethra $D_{50}$ (Gy)	<42	41.4	41.6	41.6 (41.3–42.3)	85.7	41.8 (41.5–42.3)	78.4
Rectum $D_{50}$ (Gy)	<18.1	3.7	5.2	3.7 (3.5–4.0)	100	5.2 (4.9–5.5)	100
Rectum $D_{20}$ (Gy)	<29	10.3	11.8	10.3 (8.9–12.0)	100	11.9 (10.5–13.6)	100
Rectum $D_{2cm^{3}}$ (Gy)	<36	30.2	31.0	30.2 (26.2–33.9)	99.3	31.0 (27.2–34.4)	98.9
Bladder $D_{10cm^{3}}$ (Gy)	<37	28.1	29.2	28.3 (24.9–31.7)	100	29.2 (25.9–32.5)	100
Bladder $D_{40}$ (Gy)	<18.1	4.5	5.1	4.3 (3.5–5.3)	100	4.9 (4.0–6.0)	100

Although we did not find a large difference in OAR or target dose for C and NC plans, we did find a difference in the lower dose regions. The ratio between the $V_{25}$ and $V_{50}$ was 4.7 vs. 4.1 for C and NC sparing dose distributions respectively, showing a significant dose reduction in these lower dose regions $\left(p<0.001\right)$ for NC plans.

## 4 Discussion

In this paper, we investigated the impact of NVB sparing in hypofractionated radiotherapy of prostate cancer in the presence of realistic treatment uncertainties. PCE was used to model the impact of these uncertainties and their effect on clinically relevant DVH parameters, both for the target and the OARs. To the best of our knowledge this is the first report of its kind showing that NVB sparing is maintained when accounting for realistic treatment uncertainties.

Plans were generated with a maximum dose constraint of 133 % or 48 Gy, in correspondence with a prescription isodose line of 75 % according to the PACE-trial. In theory, these plans allow for steepest dose gradients outside the targets and maximum sparing of the NVB. However, as a consequence of this approach, the urethra does require a dose constraint to obtain clinically acceptable treatment plans. In sparing plans this dose constraint is pushed to the maximum limit to achieve NVB sparing and as a consequence this mostly limits the $D_{0.1cm^{3}}$ in the NVB. Notably we surpass the constraint in the non-sparing plan as well, indicating that this is not inherently due to the sparing of the NVB. This shows that urethra dose remains a concern in complex plans, even in a non-sparing situation, that needs to be addressed to avoid urinary toxicity [39].

Previous studies have established the efficacy and safety of hypofractionation for prostate cancer. Previously, Craig et al. [40] has confirmed that the increase of tumor control probability (TCP) by hypofractionation schemes remains intact even when considering treatment uncertainties, while also ensuring that OAR constraints are met. However, the conclusions were drawn from a limited sample size of 500 simulated treatments for 10 patients, constrained by the computational demands of the Monte Carlo method. In our study we obtained similar results in terms of tumor coverage and OAR dose. By employing PCE, our study overcomes this limitation by simulating 100,000 treatments, allowing a more robust statistical analysis.

In contrast to the commonly used PTV-based evaluation approach, which assumes that the shape of the dose distribution remains unchanged by positional shifts, PCE offers a more accurate representation of treatment uncertainties in hypofractionated treatments. Traditional PTV evaluations are typically valid for fractionated treatments with minimal modulation, as they rely on the so called “static dose cloud approximation”. In highly modulated techniques, such as those used for hypofractionated prostate cancer treatments with NVB sparing, this assumption might not hold [41]. Therefore, we employed PCE, which allows for the generation of a wide range of error scenarios by sampling different combinations of positioning errors. This approach provides a more precise evaluation of how uncertainties affect tumor coverage and OAR sparing. To the best of our knowledge, this study represents the first comprehensive assessment of the impact of treatment uncertainties on hypofractionation for prostate cancer, offering statistically significant insights.

In line with the clinical practice in our institute, plans were generated with a 3 mm CTV-PTV margin [36]. To obtain statistically accurate results on probability distributions of clinically relevant DVH parameters, realistic error distributions consistent with that margin were used with systematic and random (1 SD) errors both equal to 0.94 mm. Our results confirm that adequate CTV dose is achieved in the presence of these errors, highlighting the applicability of the Van Herk margin recipe to this hypofractionated prostate treatment. PTV dose is obviously compromised, as the PTV was merely introduced in (nominal) treatment planning to achieve adequate CTV dose in the presence of errors. The PCE evaluation is limited to global rigid shifts only. Residual rotational errors (after intra-fraction correction) in patient and beam alignment as well as anatomical deformations were not explicitly taken into account. However, to first order, both rotations and deformation locally correspond to translations and are adequately mitigated by the 3 mm CTV-PTV margin. We did observe a limited change in the coverage of the CTV when sparing the NVBs, suggesting that this 3 mm margin of the PTV may be too conservative. But further clinical research should confirm this before drawing any conclusions.

While the dose reduction was larger in NC plans compared to C plans, the resulting dose to the NVB did not significantly differ between the plans with the two beam arrangements. Treatment plans were optimized on fluence alone and MLC segments were not generated. However, it is possible to segment these plans without substantial quality loss [42]. Segmentation should not introduce systematic variation in the DVH parameters on the OARs or CTV [42], and is expected to have a similar effect in non-sparing and sparing plans for both beam arrangements.

While the results of this study are promising, the clinical significance remains unclear because the dose–response relationship for NVB is not yet understood. The impact of the amount of theoretical sparing achieved in this study requires validation by ongoing clinical trials such as the POTEN-C (clinicaltrials.gov: NCT03525262) and ERECT (clinicaltrials.gov: NCT04861194) [16], [18].

In conclusion, this study explored the feasibility of NVB sparing in the presence of realistic patient and delivery uncertainties. Despite these uncertainties, we successfully achieved NVB sparing in the simulated fractionated treatments for all patients, while maintaining CTV coverage. There was no significant difference in the achieved NVB dose between NC and C plans. The clinical impact of NVB sparing needs to be established by clinical trials.

## CRediT authorship contribution statement

**Roel C. Kwakernaak:** Methodology, Software, Validation, Formal analysis, Investigation, Data curation, Writing – review & editing, Visualization. **Victor J. Brand:** Methodology, Resources, Writing – review & editing. **Jesús Rojo-Santiago:** Methodology, Software, Writing – review & editing. **Femke E. Froklage:** Conceptualization, Writing – review & editing. **Mischa S. Hoogeman:** Conceptualization, Investigation, Writing – review & editing, Supervision. **Steven J.M. Habraken:** Methodology, Software, Validation, Investigation, Writing – review & editing, Visualization, Supervision. **Maaike T.W. Milder:** Methodology, Software, Validation, Investigation, Writing – review & editing, Visualization, Supervision.

## Declaration of competing interest

The authors declare the following financial interests/personal relationships which may be considered as potential competing interests: The authors declare research collaborations with Varian Medical Systems a Siemens Healthineers Company, USA; Accuray, USA; Elekta AB, Stockholm; Siemens Healthineers, RaySearch, Stockholm, Sweden. This research project was not part of any of these collaborations.

## Appendix A Supplementary data

Supplementary Data 1 Typical Probabilistic DVH (left) for 100.000 different scenarios for both sparing and non-sparing treatment plans (coplanar) of one of the NVBs for a single patient, the median (solid line), 5th and 95th percentile (bands) of the PCE distribution are plotted as well as the nominal scenario (dashed line). Histograms (right) of the distribution of the NVB D_{0.1 cm^3}.
